# Optimal stimulation parameters for spinal and corticospinal excitabilities during contraction, motor imagery and rest: A pilot study

**DOI:** 10.1371/journal.pone.0235074

**Published:** 2020-06-22

**Authors:** Amandine Bouguetoch, Sidney Grosprêtre, Alain Martin

**Affiliations:** 1 Cognition, Action and Sensorimotor Plasticity [CAPS], INSERM, University of Bourgogne Franche-Comté, Dijon, France; 2 EA-4660 C3S Culture Sport Health Society, University of Bourgogne Franche-Comté, Besancon, France; Universita degli Studi di Catania, ITALY

## Abstract

**Objectives:**

It is commonly accepted that motor imagery (MI), i.e. the mental simulation of a movement, leads to an increased size of cortical motor evoked potentials (MEPs), although the magnitude of this effect differs between studies. Its impact on the spinal level is even more variable in the literature. Such discrepancies may be explained by many different experimental approaches. Therefore, the question of the optimal stimulation parameters to assess both spinal and corticospinal excitabilities remains open.

**Methods:**

H-reflexes and MEPs of the triceps surae were evoked in 11 healthy subjects during MI, weak voluntary contraction (CON) and rest (REST). In each condition, the full recruitment curve from the response threshold to maximal potential was investigated.

**Results:**

At stimulation intensities close to the maximal response, MEP amplitude was increased by CON compared to REST on the triceps surae. No effect of the different conditions was found on the H-reflex recruitment curve, except a small variation beyond maximal H-reflex in the soleus muscle.

**Conclusion:**

Based on our results, we recommend to assess corticospinal excitability between 70% and 100% of maximal MEP intensity instead of the classical use of a percentage of the motor threshold and to elicit H-reflexes on the ascending part of the recruitment curve.

## Introduction

Mentally simulating a movement without concomitant motor output is a cognitive activity called motor imagery [MI] and can be experienced by most people [1; 2; 3]. It has been shown that MI and actual movement share similar neural correlates [4; 5; 6; 7]. Indeed, this has been evidenced by functional magnetic resonance imaging (fMRI) studies that have shown that primary motor areas, parietal cortices, and the cerebellum, which are normally engaged in the actual execution of movements, are also activated during MI [3; 8; 9; 10; 11; 12]. Likewise, the involvement of the primary motor cortex during MI has also been evidenced by the increased size of motor evoked potentials (MEPs), induced by transcranial magnetic stimulation (TMS), as compared to rest [13]. The magnitude of this MEP change, reflecting an increase of corticospinal excitability, is of various amount from one study to another [14]. These differences may be for a great part attributed to the large range of TMS intensities from motor threshold to 100% of the stimulator output used in the different studies [15; 16; 17]. To assess corticospinal excitability, it has been recently recommended to assess the full recruitment curve of the MEP rather than recording one stimulation intensity [18].

While variable, the corticospinal excitability modulation during MI is well established. On the contrary, the involvement of spinal mechanisms is more controversial. Indeed, conflicting results regarding the effects of MI on spinal excitability have been reported. Most studies used an H-reflex technique, i.e. measuring the myoelectrical response resulting from Ia afferent fibers activated by percutaneous peripheral nerve stimulation. Actually, it is the most commonly used tool to assess modulation of spinal excitability [19]. Some studies showed an increase of H-reflex amplitude during MI compared to rest [15; 20] while several other studies showed that MI had no significant influence [13; 16; 17; 21; 22; 23]. As for the behavior of MEPs, these discrepancies may mainly arise from the methodology used to evoke H-reflex responses. No spinal excitability modulation has been observed during MI by Hashimoto and Rothwell [13] for an H-reflex amplitude of 50% of the maximal H-reflex on the flexor carpi radialis muscles (FCR) while Bonnet et al. in 1997 [20] using an H-reflex amplitude ranging from 25% to 50% of the maximal H-reflex in the posterior tibial nerve have reported an increase in spinal excitability during MI. Beyond the methodological aspects, the inconsistency in the results of these studies could be related to the fact that the effect of MI on spinal excitability could depend on the initial H-reflex size. Recommendations have been made during the last decades concerning the optimal stimulation parameters to elicit a reliable H-reflex among different muscle activation conditions [24]. As a matter of fact, when comparing active (i.e. actual contraction) and resting states, Grosprêtre and Martin [25] showed that the reliability of the H-reflex seems to be higher when considering values prior to the maximal H-reflex amplitude, i.e. in the ascending part of the recruitment curve.

Therefore, while some recommendations have been established for corticospinal and spinal excitability assessment from rest to contraction (or ‘passive to active’), no such consensus has been established for MI studies. Importantly, these considerations should not necessarily apply to MI, since it has not been established if its neural activation can be considered as an active, a passive, or an intermediate condition. However, some studies reported primary motor cortex activation during MI equivalent to approximately 30% of the level observed during actual execution [8; 26]. In response to these observations, the first aim of our study was to assess the effects of MI on spinal and corticospinal excitabilities in comparison to both voluntary contraction (CON) and rest conditions (REST) by building complete MEP and H-reflex recruitment curves in the triceps surae. This would help to establish strong recommendations regarding stimulation parameters to apply when comparing spinal and corticospinal excitability during MI to those during actual contraction or rest. Finally, the comparison of MEP and H-reflex recruitment curves at rest, during MI and slight contraction, while only two of those three conditions are usually assessed, could also help question the theory that MI represents an intermediate neural activation between passive and active states.

## Methods

### Participants

Twelve young healthy subjects (age: 25.5±8.5 years old; height: 176.5±6.9 cm; weight: 71.8±12.9 kg, 2 females) gave written informed consent to participate in the present study. Main exclusion criteria were age under 18 and reporting of any neurological or muscular disorders. Each participant completed the revised version of the Movement Imagery Questionnaire [27] to determine self-estimation of MI ability. The mean MIQ-R score was 47 ± 4 (maximum score: 56), indicating a good imagery capacity. They were requested to avoid any intense exercise prior the experimental session. Sample size calculation was based on previous experiments about the effects of MI on some of the tested variables [28, 29]. Considering a significance level of 5% and a power of 90%, a minimum of 10 participants was requested to meet the objectives of the study. The experimental design was approved by the regional ethic committee (CPP COOM III number 2017-A00064-49; Clinical trial.gouv identifier NCT03334526) and conducted in conformity with the latest version of the Declaration of Helsinki.

### Experimental design

**Fig 1:**
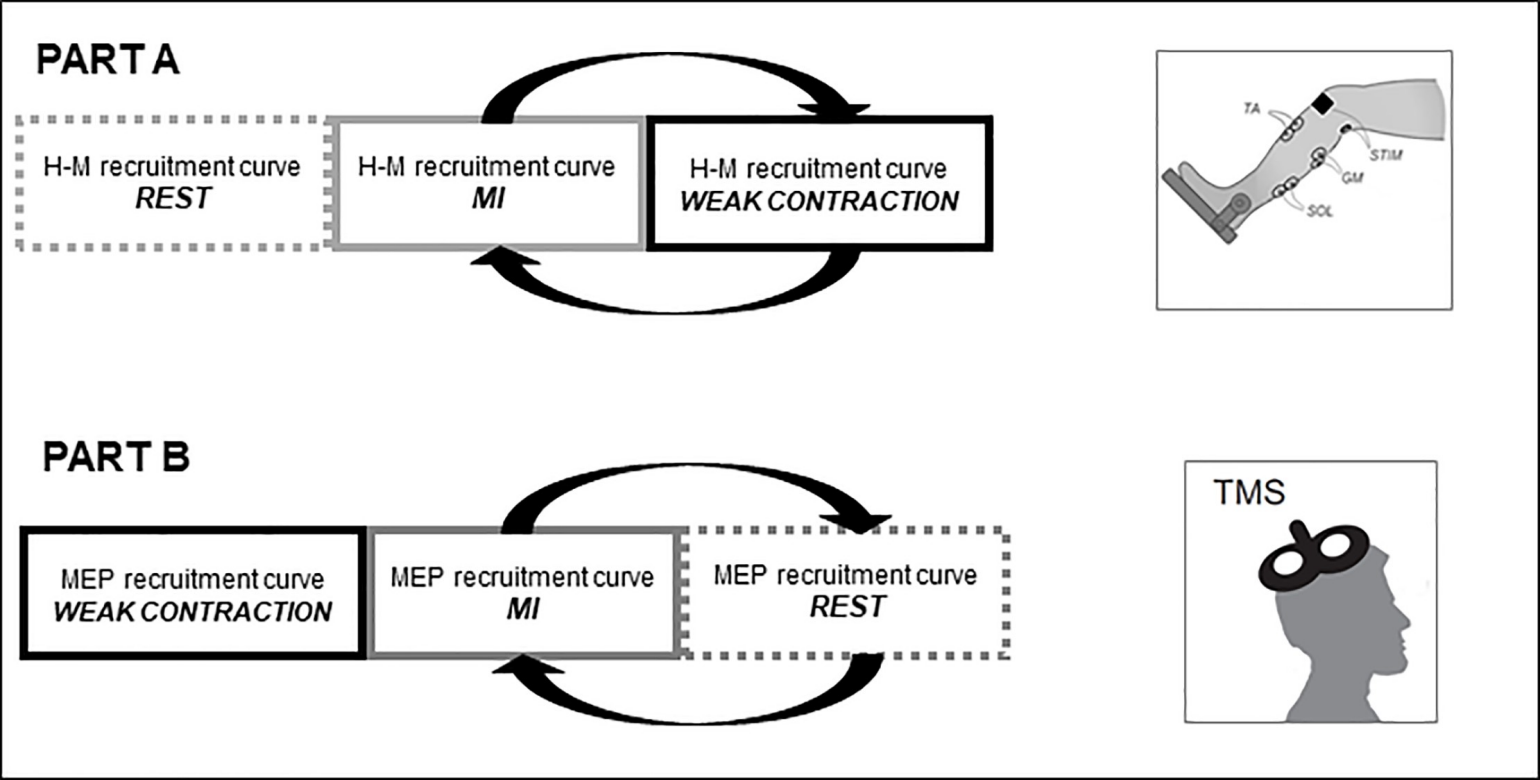
Experimental protocol. TMS: transcranial magnetic stimulation. MEP: motor evoked potential. MI: motor imagery. ISI: inter-stimuli interval. The arrows express randomization between the conditions.

All participants performed one experimental session of about 2 hours on their right calf, involving 2 different parts (Fig 1).

For each part, A or B, stimulations were applied under 3 conditions: at rest (REST), during contraction (CON) and during MI. Part A consisted in recording H-M recruitment curves of triceps surae muscles from the H-reflex threshold to the maximal muscle compound action potential (M_max_). CON and MI were performed randomly.

In part B, MEP recruitment curves were recorded in the same muscles. The first condition carried out was CON, for which it is easier to find the stimulation site and then the two other conditions were performed in a random order.

During the whole session, participants seated on an isokinetic chair [Biodex system 4, Shirley, NY] with their right foot firmly tied on a pedal and set at a 90°-ankle angle (angle measured between the tibia and the sole of the foot), with the motor axis aligned with the external malleolus of the ankle. Knee and hip angles were also set at 90°. Myoelectrical activities (EMG) of the right soleus muscle (SOL), medial and lateral gastrocnemii muscles (MG and LG) and tibialis anterior muscle (TA) were recorded throughout the session.

First, participants were asked to warm-up their calf muscles by pushing sub-maximally against the pedal ten times with a force increment. Then, participants were asked to perform at least two maximal voluntary contraction (MVC), separated by two minutes’ rest. Further trials were performed if the variation in maximal performance between these trials exceeded 5%.

During REST, the participants were asked to stay still. To ensure that the targeted muscles were totally relaxed, background EMG activity was monitored. During MI and CON, an auditory signal was given to the participants randomly between 0.5 and 2 seconds before each stimulation to start imagining or contracting to avoid any anticipation of the stimulation and another 2 seconds after the stimulation to stop the task. During MI conditions, participants were instructed to imagine pressing maximally on the pedal [MI of maximal isometric plantar flexors contraction] and to feel the corresponding muscle contraction [kinesthetic MI]. During actual contraction, participants were able to reach and maintain a slight voluntary contraction of 5% MVC by means of a monitor displaying the torque signal placed in front of them.

### Electromyography [EMG] recording

EMG activity was recorded from four muscles of the leg (SOL; MG; LG; TA). After shaving and dry-cleaning the skin with alcohol to keep low impedance (< 5 kΩ), EMG signals were recorded by using two silver-chloride surface electrodes (8mm diameter) placed at an interelectrode center-to-center distance of 2 cm. For SOL, electrodes were placed 2 cm below the insertions of the gastrocnemii over the Achille’s tendon; for MG and LG, over the mid belly of the muscles; and for TA, at 1/3 of the distance between the fibula and the medial malleolus. A common reference electrode was placed between stimulation and recording sites. EMG signals were amplified with a bandwidth frequency ranging from 15 Hz to 5 kHz (gain = 1000) then digitized on-line (sampling frequency: 5 kHz) using the MP150 Biopac system and stored for analysis with Acqknowledge software 4.2.

### Nerve electrical stimulation

Posterior tibial nerve stimulations were used to record H-reflexes and M-waves of the considered muscles. Single rectangular pulses (1-ms width) were delivered by a high voltage (400 V) constant-current stimulator (Digitimer model DS7A, Hertfordshire, UK), through a self-adhesive cathode [8-mm diameter, Ag-AgCL) placed in the popliteal fossa and an anode (5 x 10 cm, *Medicompex SA*, *Ecublens*, *Switzerland*] placed over the patella. Optimal stimulation site was first located by a hand-held cathode ball electrode (0.5-cm diameter) in order to obtain the greatest H-reflex amplitudes for the lowest stimulation intensity. Measures were optimized for the soleus muscle. Particular care was taken in avoiding EMG responses on the antagonist muscle (TA). Once determined, stimulation electrodes were firmly fixed to the optimal site with straps.

To build H-M recruitment curves, stimulations started at the H-reflex threshold and were progressively increased with a 4-mA increment until the maximal M-wave amplitude (M_max_) was reached. To ensure that the M-wave lied in the plateau of its maximal value, maximal stimulation intensity was increased by 20%. Subsequently, 2 mA-increments were performed around maximal H-reflex to accurately estimate its intensity. Three responses were evoked at each intensity, with an inter-stimuli interval of 10 seconds.

### Transcranial magnetic stimulation [TMS]

To elicit MEPs, a double-cone coil was positioned over the contralateral [left] primary motor cortex and connected to a stimulator (Magstim 200, Magstim Company Ldt, Great Britain). The optimal stimulation site for the soleus muscle was found by placing the coil over the left motor cortex, and stimulating from 1 cm posterior and lateral to the vertex of the subject's head. Optimal stimulation site was identified during a 5%-MVC-plantar flexion and marked on a bathing cap worn by the participants. The coil was secured by using a tripod with a lockable articulated arm in order to deliver antero-posterior current to the brain. Given the weak contraction level, active motor threshold (aMT) was defined as the minimal TMS intensity required to evoke MEP peak-to-peak amplitudes greater than 50 μV in the targeted muscle in 3 out of 5 consecutive trials.

MEP recruitment curve was built during voluntary contraction at 5%MVC, from the aMT to the maximal MEP amplitude, with an increment of 5% of the maximal stimulator output. Three trials were recorded at each intensity with a 10-second inter-stimuli interval.

Afterward, MEP recruitment curves during MI and REST were recorded in a random order starting from the highest intensity reached during the contraction condition (different among subjects) and decreasing every 5% of the stimulator output. The measures were stopped as soon as a MEP was no longer elicited, i.e. reached amplitudes and occurrences below the threshold (i.e. less than half of the responses of at least 50 μV amplitude). Given the hypothesis that the rest threshold would be higher than active threshold, starting from the highest TMS intensity obtained during CON allowed to limit the number of stimulations that the subjects had to undergo. The thresholds of the several conditions [MI, REST and CON] were noticed and considered further analysis.

## Data analysis

Peak-to-peak amplitudes of each electromyographic responses (M-waves, H-reflexes and MEP) were measured and averaged among the different trials. The amplitude of the submaximal M-wave accompanying each H-reflex, noted M_atH_ was also taken into consideration. The root mean square (RMS) of EMG signal of each muscle was measured over the 500ms preceding each stimulation artifact during MI and REST to ensure that participants stayed relaxed [30].

### Recruitment curves

In order to build recruitment curves for each parameter, all the H-reflexes, M-waves and MEP values were normalized by the maximal M-wave of the corresponding muscle and condition (H/M_max_; M_atH_/M_max_; MEP/M_max_) and plotted against their corresponding intensity, expressed as a percentage of maximal intensity (i.e. M_max_ intensity).

While SOL was analyzed separately, H-M and MEP recruitment curves of the gastrocnemii muscles were built by summing the raw values of MG and LG muscles and normalizing them by the sum of their M_max_ values. To assess the triceps surae as a whole, raw values of the three muscles were summed and then normalized by the sum of M_max_ values. Indeed, GM and GL are two parts of the same muscle with an anatomical function which is different from SOL and the triceps surae is the functional muscle group of the calf, innervated by the tibial nerve. Mean recruitment curves over all participants were built by sorting out the H-M values by ranges of 10%M_max_ intensity and MEPs by ranges of 10% of maximal intensity. The average of all the participants in each range was used for statistical analysis performed on the recruitment curves.

### Statistical analysis

All data are presented as the mean ± standard deviation. A Shapiro-Wilk test (p<0.05) was used to test the normality. SOL, MG+LG and the whole triceps surae were analyzed separately for the H-M and MEP recruitment curves.

A repeated-measures ANOVA was used on normalized responses to assess the within effects of condition (MI, CON and REST), stimulation intensity and interaction between the two. When a main or interaction effect was found, a post-hoc analysis was conducted using Tukey’s HSD (honest significant difference) test. Effect size is indicated in the results using partial eta square (ηP^2^).

A one-way ANOVA with the factor “condition” (MI, REST, CON) was also used on normalized responses to compare the effect of conditions, one range of intensity at a time.

Statistical analysis was performed with Statistica software (10.0 version, Statsoft, Tulsa, Oklahoma, USA). The level of significance was set at p < 0.05.

The interpersonal variability of response to the condition on MEPs amplitude was expressed as coefficients of variation (CV) calculated for SOL, MG+LG and triceps surae as (standard deviation/average)*100 for each intensity interval. Then, these results were averaged for the whole condition.

## Results

### Motor evoked potentials

**Fig 2:**
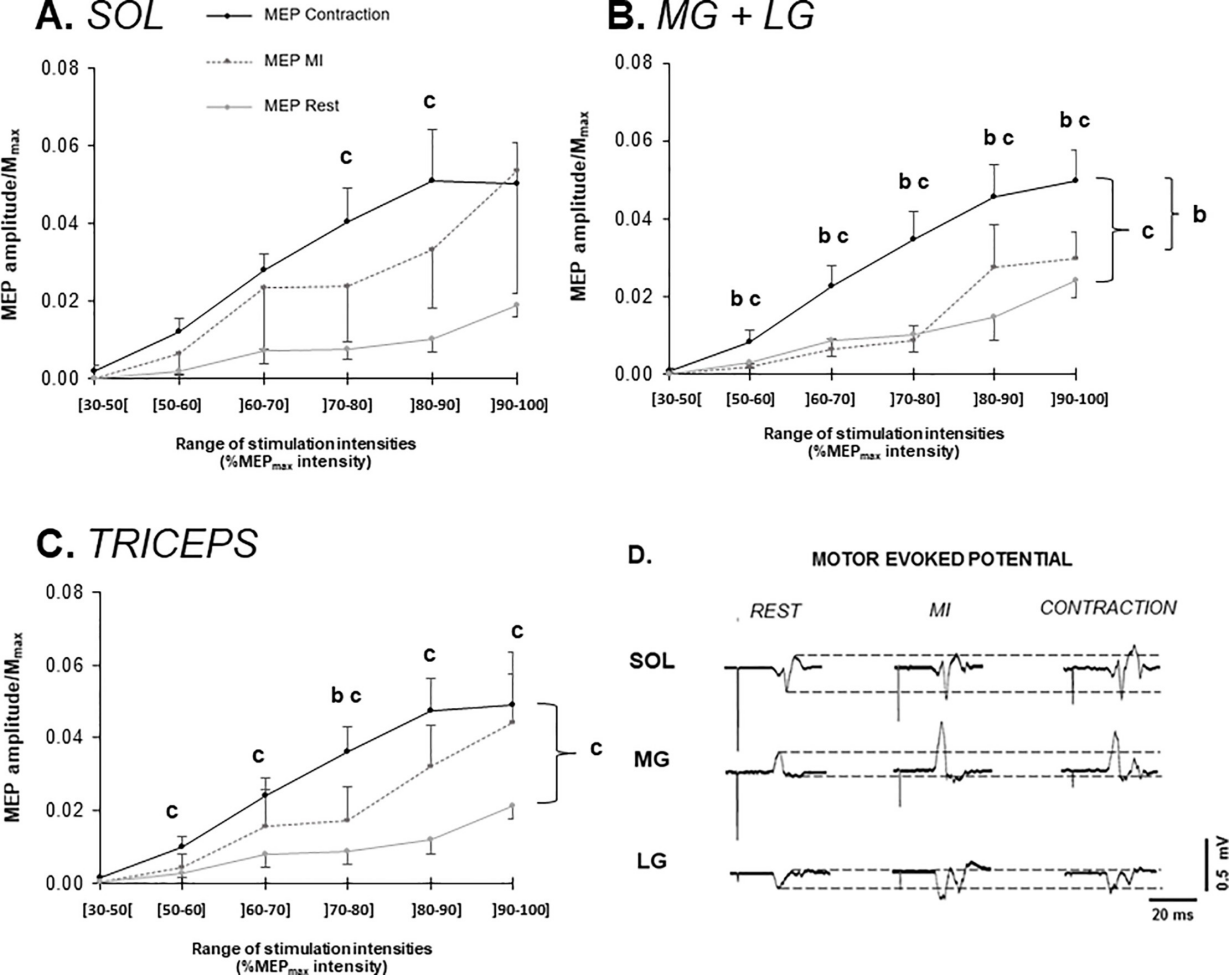
MEP peak-to-peak amplitudes normalized by M_max_ in all three conditions for the soleus muscle [A], the gastrocnemius muscles [B] and the triceps surae [C] are displayed as mean ± SEM. In the bottom right corner, typical EMG recordings of MEP for each muscle and each condition [D]. The dotted lines indicate the peak-to-peak amplitude at rest. b: Statistical difference between CON and MI, at P<0.05. c: Statistical difference between CON and REST, at P<0.05.

The ANOVA comparing MEP/M_max_ between the three conditions showed no difference for SOL (p = 0.12; F[2,20) = 2.4; ηP^2^ = 0.19). But there was a significant intensity effect (p≤0.001; F(5,50) = 12.1; ηP^2^ = 0.55). The ANOVA comparing all the conditions intensity by intensity showed a significant difference between CON and REST for the ranges from 70% to 90%MEP_max_ intensity (p≤0.031; ηP^2^ = 0.28 and 0.29) (Fig 2A).

For MG+LG, the ANOVA showed that MEP/M_max_ were statistically higher during CON than REST (p = 0.0013; ηP^2^ = 0.51) and MI (p = 0.0042; ηP^2^ = 0.51). The ANOVA also revealed a significant intensity effect (p≤0.001; F(5,50) = 23.6; ηP^2^ = 0.7). The interaction effect showed more specifically that these higher values during CON were present from 50 to 100%MEP_max_ intensity (Fig 2B).

When the whole triceps surae was considered, MEP/M_max_ were also statistically higher during CON than REST (p = 0.016; ηP^2^ = 0.32). A statistical difference was also observed between CON and MI for the range from 70% to 80%MEP_max_ intensity (p = 0.049; ηP^2^ = 0.42) (Fig 2C).

The effect of MI on MEPs amplitude varied among participants, this interpersonal variability of response to MI being expressed by mean CV for all intensity intervals: 204.1% Triceps, 230.1% SOL, 162.8% GM+GL. CV assessing interpersonal variability to CON were 97.2% Triceps, 100.5% SOL and 103.4% MG+LG. It can be noted, considering pooled data for each condition, that the CV were higher for MI than CON.

### H-M recruitment curves

**Fig 3:**
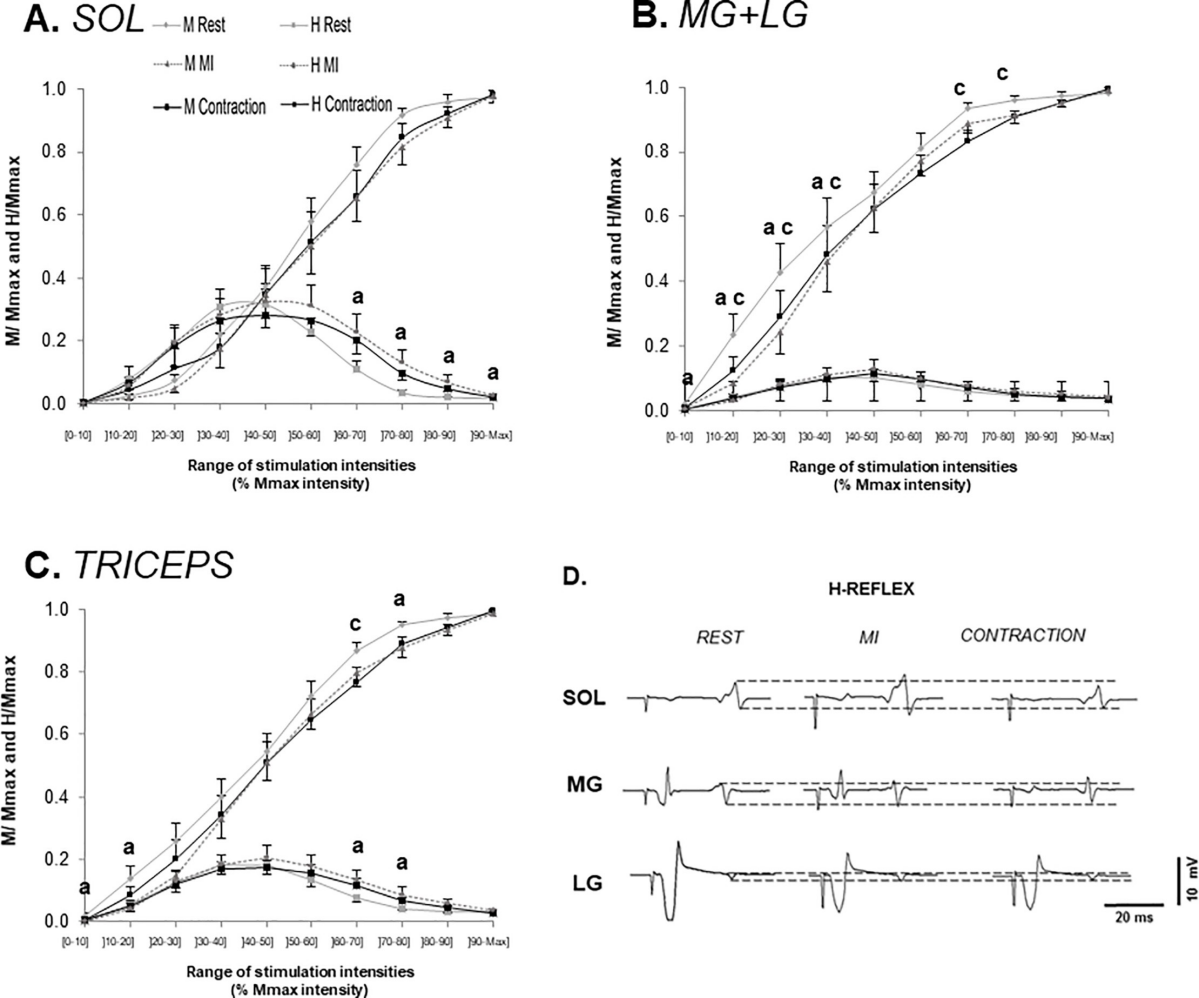
H-M recruitment curves in all three conditions for the soleus muscle [A], the gastrocnemius muscles [B] and the triceps surae [C]. M-waves and H-reflexes’ peak-to-peak amplitudes normalized by M_max_ are displayed as mean ± SEM. In the bottom right corner, typical EMG recordings of H-reflexes for each muscle and each condition [D]. The dotted lines indicate the peak-to-peak amplitude at rest. a: Statistical difference between REST and MI, at P<0.05. b: Statistical difference between CON and MI, at P<0.05. c: Statistical difference between CON and REST, at P<0.05.

The ANOVA showed no effect on the three conditions on the M-wave recruitment curves for SOL (p = 0.29; F(2,22) = 1.3; ηP^2^ = 0.11) but it showed an intensity effect (p≤0.001; F(9,99) = 118.4; ηP^2^ = 0.92) for every condition (Fig 3A). Maximal M-waves amplitudes were not significantly different between the conditions (REST: 10.5 mV±4.1; CON: 10.7 mV±4.0; MI: 10.5 mV±4.1).

The MG+LG M-waves [Fig 3B) at REST were significantly different from CON (p = 0.039; ηP^2^ = 0.3) and MI (p = 0.035; ηP^2^ = 0.3). The ANOVA also showed an intensity effect [p≤0.001; F[9,99) = 90.2; ηP^2^ = 0.89). The interaction effect (p = 0.015; F(18,198) = 1.94) showed more specifically a significant difference between REST and CON for the ranges from 10% to 40%M_max_ intensity (p≤0.047; ηP^2^ = 0.15) and 60% to 80%M_max_ intensity (p≤0.044). REST was also different from MI between 0% and 40%M_max_ intensity (p≤0.038; ηP^2^ = 0.15). Maximal M-waves amplitudes were not significantly different between the conditions (REST: 8.4 mV±5.7 GM and 8.0 mV±4.0 GL; CON: 8.6 mV±5.5 GM and 8.5 mV±4.1 GL; MI: 8.7 mV±5.9 GM and 8.1 mV±4.1 GL)).

The ANOVA showed no significant condition effect on the triceps surae M-waves (Fig 3C) (p = 0.052; F(2,22) = 3.4; ηP^2^ = 0.24) but it revealed an intensity effect (p≤0.001; F(9,99) = 172.0; ηP^2^ = 0.94). The ANOVA comparing all the conditions intensity by intensity showed a significant difference between REST and MI for the ranges 0–10 and 10–20%M_max_ intensity (p≤0.042; ηP^2^ = 0.23 and 0.21) and 70–80%M_max_ intensity (p = 0.041; ηP^2^ = 0.23). It also showed a significant difference between REST and CON for the range 60–70%M_max_ intensity (p = 0.021; ηP^2^ = 0.25).

The ANOVA on the H-reflex recruitment curve of SOL showed no difference between the conditions (p = 0.21; F(2,22) = 1.7; ηP^2^ = 0.13) but an intensity effect existed (p≤0.001; F(9,99) = 14.2; ηP^2^ = 0.56). However, the ANOVA comparing all the conditions intensity by intensity showed a significant difference between REST and MI for the ranges from 60% to 100%M_max_ intensity (p≤0.039; 0.21≤ηP^2^≤0.28). This significant decrease of the H-reflexes amplitudes at REST was correlated to the non-significant increase of the M-waves amplitudes accompanying the H-reflexes in the same condition (R^2^ = 0.74). This was calculated by subtracting the values of the H-reflex at rest to the values during MI for each intensity range. The same was done to the M-waves values.

The ANOVA concerning MG+LG showed no difference between the conditions (p = 0.40; F(2,22) = 0.97; ηP^2^ = 0.08) but a significant intensity effect (p≤0.001; F(9,99) = 8.7; ηP^2^ = 0.44).

For the triceps surae, the statistical analysis showed no difference between the conditions (p = 0.22; F(2,22) = 1.6; ηP^2^ = 0.13) but a difference between the intensities (p≤0.001; F(9,99) = 12.9; ηP^2^ = 0.54). However, the ANOVA comparing all the conditions intensity by intensity displayed a significant difference between REST and MI for the ranges from 60 to 70 and 70 to 80%M_max_ intensity (p≤0.036; ηP^2^ = 0.25 and 0.21).

## Discussion

The present study showed that higher values of MEPs were found in CON as compared to REST, while no effects between MI and REST nor between MI and CON were found (Fig 2). No effect of condition was found on H-reflex’s amplitudes in the ascending part of the recruitment curves for all tested intensities (Fig 3), while an effect was found on the descending part of the soleus curve, as H-reflex during MI was statistically higher than at rest in this portion. This was also reflected on the triceps surae curve.

### Effects on corticospinal excitability

The particularity of the present study was that responses were tested during three different potential level of activation of the corticospinal tracts. A difference was found between CON and REST that started to be statistically relevant at 70% of MEP_max_ intensity. In line with the literature [19], this result indicates an increase of the corticospinal excitability as soon as the muscle is voluntarily contracted.

Interestingly, MEP/M_max_ during MI was not different from either REST or CON conditions on SOL and the triceps surae. Thus, MI recruitment curve was placed in between those two extremes (Fig 2A and 2C), suggesting that MI could be an intermediate level of activation of the corticospinal pathway. The involvement of the primary motor cortex during MI has already been evidenced by the increased size of MEP induced by TMS, as compared to rest in other studies [13; 17]. It can be argued that the presence of three conditions and many stimulation intensities, therefore representing a large amount of data, prevented MI to be statistically different from either REST or CON. Indeed, the effect of MI on MEPs amplitude varied among participants, being either closer to REST than to CON or vice-versa. This interpersonal variability of response to MI (coefficients of variation: 204.1% Triceps, 230.1% SOL, 162.8% GM+GL) caused a large standard deviation that may have drowned any effect of MI, especially on the soleus muscle, although CON was statistically different from REST. No difference was found in MIQR results between participants (low inter-individual variations), which means that this variability could not be attributed to their estimated ability to imagine movements, at least according to the tool used in the present study.

As a result, our present results revealed that MEP recruitment curve during MI fits between REST and CON recruitment curves, which tended to confirm the hypothesis stating that MI activation is intermediate between those observed at rest and during weak voluntary contractions. To support this hypothesis, it should be noticed that MEP thresholds differed from one condition to another, in the following order: CON < MI < REST. This explained why differences between conditions appeared only at relatively high TMS intensities.

In fact, in the different studies using TMS, two methods can be highlighted. Temesi et al. [31] noticed that researchers use either i) TMS intensity of 120–130% of the rest (rMT) or active (aMT) motor threshold, or ii) TMS intensity determined using stimulus-response curves during muscular contraction [32;33]. To support this latter method, Groppa et al. in [34] stated that TMS should use an optimal stimulus intensity that marks the transition from the rising slope to the plateau of the sigmoid recruitment curve. If the target muscle is preactivated, it can be assumed that the transition from the steeper part of the slope to the plateau of the stimulus-response curve occurs at a stimulus intensity of 140% of rMT (corresponding to approximately 170% of aMT). In our study, since the intensity needed to evoke a maximal MEP did not change across the conditions, motor thresholds corresponded to values of intensity ranging from 30 to 90% of MEP_max_ intensity. Thus, using rest or active motor thresholds as a reference point is probably not accurate enough and the measures based on one of them would not place the MEP response on the same part of the recruitment curve. These observations argue in favor of setting a TMS intensity by using MEP_max_ as a reference point instead of MT, in accordance with the statement of Groppa et al [34]. In the present study, the difference between conditions started to be observed at 70% of MEP_max_. Therefore, this intensity could be high enough to be used as a reference. On the contrary, using the maximal MEP could taint the results by provoking a threshold effect which would prevent any facilitation to appear. This emphasizes the fact that when more than 2 conditions are compared (REST and MI or CON), it could be recommended to use TMS intensity in between 70% and 90% of maximal MEP intensity.

Then, the increase of corticospinal excitability seemed to be muscle-dependent according to the imagined task. Indeed, MEP/M_max_ on gastrocnemii were significantly different between CON and MI and CON and REST while they were not between MI and REST, suggesting that MI and REST may be two similar states here. This denoted a different behavior between SOL and gastrocnemii (Fig 2), MEP modulations during MI being more important on SOL. This should indicate that the effect of MI could be targeted to SOL when participants are asked to imagine a maximal isometric plantar flexion. This could be explained by several factors, for example gastrocnemii also acting as knee flexors while SOL is monoarticular around the ankle. However, the aforementioned intermediate modulation of MEPs during MI observed on SOL was also found when compiling all triceps surae muscles, indicating that the global behavior of this muscle group leans toward MI being an intermediate state. Again, the large inter-individual variability in the way to activate either the corticospinal tract of SOL only or also those of gastrocnemii during MI may account for this result. From a methodological point of view, this emphasizes the interest to assess such muscle groups as a whole when assessing MI task modulations.

Taken together, the present results on MEP recruitment curves argue in favor of what can be called the continuum hypothesis, i.e. MI reflects a corticospinal activation in a continuum between rest and voluntary contraction. This means that the same motor pathways are activated during imagined and actual contractions, but into a lower extent during MI. Some studies have shown more precisely that primary motor cortex activation reported during MI amounts to only ~30% of the level observed during execution [8; 26]. The magnitude of imagery-induced cortical activity was ∼25% that of actual movement according to a study of Miller et al in 2009 [35] using electrocorticography. As a matter of fact, there is another theory to the neural correlates of MI. It postulates that motor output would be blocked before it reaches the motoneuron level, by an inhibitory mechanism generated in parallel to motor activation [5]. However, the present study as well as previous ones demonstrating that MI and CON share similar neural pathways argue in favor of a lack of inhibition or at least an incomplete inhibition, favoring the continuum hypothesis. MI seems to trigger a sub-threshold activation of corticospinal tracts which could partly involve spinal networks [28, 36]. As a consequence, MEPs being representative of the motor pathway from the brain to muscles, it is then relevant to investigate the impact of this increased corticospinal excitability on the spinal level to identify the relative contributions of cortical and spinal levels.

### Effects on spinal excitability

The Hoffmann reflex (H-reflex) reflects transmission efficacy of Ia afferent input addressed to motor neurons at spinal level [37]. As noted in the introduction, conflicting results regarding the effects of MI on H-reflex amplitudes have been reported. In the present study, no effect was found on the ascending part of H-reflex recruitment curve no matter the conditions and muscles. However, an increase in H-reflex amplitude was found from REST to MI in the descending part of the curve, i.e. beyond maximal H-reflex intensity. This is in accordance with a previous study [25] which showed no difference in H-reflex amplitude between submaximal contractions and passive conditions in the ascending part of the reflex recruitment curve. This effect was attributed to a collision in motor axons caused by nerve stimulation between the voluntary central motor command and the antidromic impulse generated by exogenic motor axons activation (generating M-wave toward the muscle). In the present study, the difference found on H-reflex of SOL in the descending part of the curve between MI and REST was correlated with a change in the associated M-wave (R = 0.861; ddl = 10). This also explains the effect between 60 and 80%M_max_ intensity on triceps surae. Accordingly, we can conclude that this methodological consideration is the cause of such H-reflex increase observed during MI. Therefore, when considering the ascending part of the recruitment curve, as it is recommended when comparing rest and contraction conditions [25], no effect of MI can be observed on spinal excitability. Our results are in accordance with most of the other studies investigating the effect of several conditions (MI vs REST or CON) on H-reflex amplitude [13; 16; 17; 21; 22; 23]. Thus, the increase in spinal excitability during other previous studies [15; 20] may raise from methodological concerns. For instance, Kiers et al. [15] noted an increase in raw H-reflex amplitude without taking into account the evolution of the concomitant M-wave nor normalizing the response.

However, H-reflexes test only a small fraction of the structures on a spinal level [13]. Thus, the absence of H-to-M_max_ modulation do not indicate a total absence of MI effect at spinal level. While H-reflex is a standard measure of the Ia-to-alpha motoneuron synapse, other spinal structures can be assessed by using H-reflex condition paradigms. Accordingly, previous experiments showed that in absence of H-reflex modulation, other spinal circuits were modulated, particularly those involving presynaptic inhibitory interneurons [25; 28]. Notwithstanding, despite a lack of modulation of H-reflex immediately during MI, spinal excitability was shown to be enhanced after a full mental training involving several sessions of maximal mental efforts [29]. This raises the interest to measure spinal excitability through an H-reflex technique, with our previous recommendations, when considering training-based approach and longitudinal studies.

### Implications

This study aimed at establishing recommendations regarding stimulation parameters to apply when comparing spinal and corticospinal excitability during MI to those during actual contraction or rest. The first methodological advice is to measure MEP between 70% and 90% of MEP_max_ intensity instead of basing the intensity on the motor threshold, which changes from one condition to another. In fact, this usually places the measures [120%MT] in the early part of the recruitment curve where no effect of the condition is visible yet. This study also reinforces the fact that measures of the H-reflex must be done on the ascending part of the recruitment curve where the amplitude is not affected by stimulation conditions and with a small accompanying M-wave to still ensure that the stimulation conditions have not changed.

### Conclusion

To conclude, this study has also demonstrated that MEP is modulated by voluntary contraction but also by MI which might represent an intermediate level of activity between contraction and rest.

On the other hand, there seems to be no effect of the condition on H-reflex which reflects only one small part of the spinal modulations. Investigating the effects of MI on structures with a lower level of excitability such as presynaptic inhibitory interneurons could give more clues about how the spinal level is impacted by MI.

Finally, the present results argue in favor of the existence of a continuum of neural activation from rest to MI and from MI to actual contraction. At least, MI could represent a subthreshold activation of the neural structures that are shared with actual contractions. Although MI of several force levels seems not to have a different effect on MEPs [38], further experiments assessing several levels of imagined and actual contraction forces with the newly proposed stimulation parameters could bring new evidence onto this continuum theory.
